# Increasing microtubule acetylation rescues axonal transport and locomotor deficits caused by LRRK2 Roc-COR domain mutations

**DOI:** 10.1038/ncomms6245

**Published:** 2014-10-15

**Authors:** Vinay K. Godena, Nicholas Brookes-Hocking, Annekathrin Moller, Gary Shaw, Matthew Oswald, Rosa M. Sancho, Christopher C. J. Miller, Alexander J. Whitworth, Kurt J. De Vos

**Affiliations:** 1 Department of Biomedical Sciences, University of Sheffield, Firth Court, Western Bank, Sheffield S10 2TN, UK; 2 The Bateson Centre, University of Sheffield, Sheffield S10 2TN, UK; 3 Centre for Membrane Interactions and Dynamics, University of Sheffield, Sheffield S10 2TN, UK; 4 Department of Basic and Clinical Neuroscience, Institute of Psychiatry, Psychology and Neuroscience, King’s College London, De Crespigny Park, Denmark Hill, London SE5 8AF, UK; 5 Department of Neuroscience, Sheffield Institute for Translational Neuroscience (SITraN), University of Sheffield, 385a Glossop Road, Sheffield S10 2HQ, UK

## Abstract

*Leucine-rich repeat kinase 2* (*LRRK2*) mutations are the most common genetic cause of Parkinson’s disease. LRRK2 is a multifunctional protein affecting many cellular processes and has been described to bind microtubules. Defective microtubule-based axonal transport is hypothesized to contribute to Parkinson’s disease, but whether LRRK2 mutations affect this process to mediate pathogenesis is not known. Here we find that LRRK2 containing pathogenic Roc-COR domain mutations (R1441C, Y1699C) preferentially associates with deacetylated microtubules, and inhibits axonal transport in primary neurons and in *Drosophila*, causing locomotor deficits *in vivo*. *In vitro*, increasing microtubule acetylation using deacetylase inhibitors or the tubulin acetylase αTAT1 prevents association of mutant LRRK2 with microtubules, and the deacetylase inhibitor trichostatin A (TSA) restores axonal transport. *In vivo* knockdown of the deacetylases *HDAC6* and *Sirt2,* or administration of TSA rescues both axonal transport and locomotor behavior. Thus, this study reveals a pathogenic mechanism and a potential intervention for Parkinson’s disease.

## Abstract (web summary)

Mutations in the kinase *LRRK2* are associated with Parkinson’s disease. Godena *et al.* find that disease-associated *LRRK2* mutations promote its binding to deacetylated microtubules, and cause defects in axonal transport and *Drosophila* locomotor behaviour that can be reversed by enhancing tubulin acetylation.

## Graphical abstract

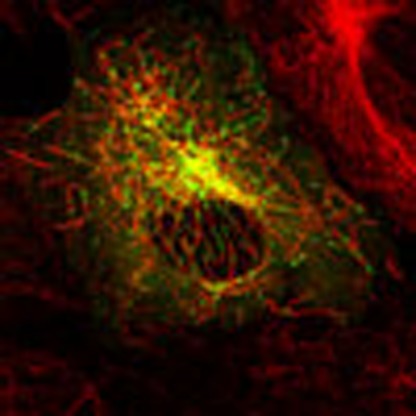

Dominant mutations in *leucine-rich repeat kinase 2* (*LRRK2*) are the most common genetic cause of Parkinson’s disease (PD), and genome-wide association studies have highlighted the *LRRK2* locus as a risk factor for sporadic PD^1^,^2^,^3^,^4^,^5^. LRRK2 is a multifunctional, multi-domain protein and dominant mutations have been identified in the Ras of complex (Roc) GTPase protein domain (R1441C, R1441G, R1441H), the carboxy-terminal of Roc (COR) domain (Y1699C) and the kinase domain (G2019S and I2020T). Mutations in the Roc and COR domains decrease GTPase activity^6^,^7^, whereas the G2019S mutation increases kinase activity^6^,^8^,^9^. How LRRK2 mutations confer toxicity is not yet clear, but may involve the possible physiological function of LRRK2 in the maintenance of axonal integrity. Indeed, there is evidence that the overexpression of LRRK2 causes neurite shortening, whereas the loss of LRRK2 function in mouse neurons results in elongation of neuronal processes and increased branching^10^,^11^,^12^,^13^. The molecular details underlying mutant LRRK2’s effects on neurites are not fully understood, but may involve interactions with microtubules^14^,^15^,^16^,^17^,^18^. The architecture of neurons makes them particularly dependent on efficient intracellular transport of cargoes such as organelles and proteins to maintain structural integrity and function. Since defective axonal transport, which utilizes microtubules as transport tracks, has been proposed as a mechanism for PD^19^,^20^,^21^,^22^,^23^,^24^, we hypothesized that mutant LRRK2 may disturb microtubule-based axonal transport.

Here we show that LRRK2 Roc-COR mutants disrupt axonal transport *in vitro* and *in vivo* to induce locomotor deficits by binding to deacetylated microtubules. Increasing microtubule acetylation prevents microtubule association, restores axonal transport and rescues locomotor deficits caused by LRRK2 Roc-COR mutants. Thus, our data reveals a pathogenic mechanism and a potential therapeutic intervention for PD.

## Results

### Roc-COR mutations inhibit axonal transport and locomotion

**Figure 1:**
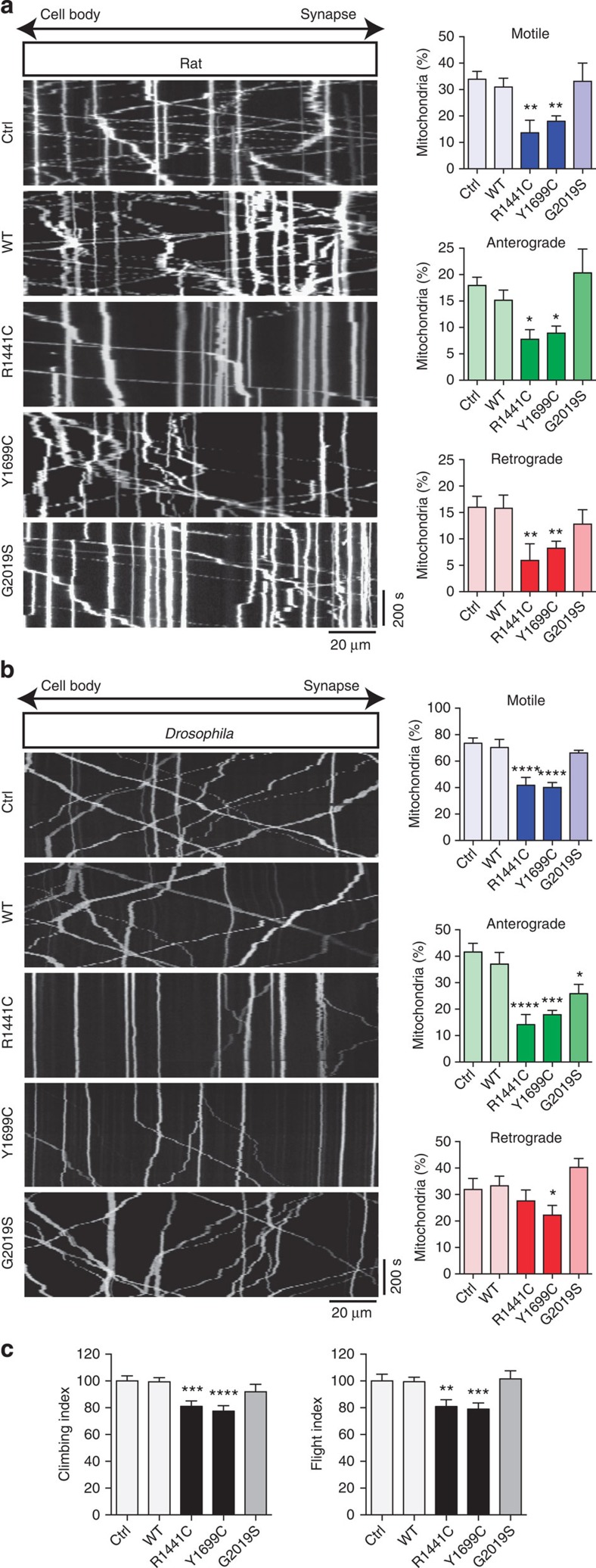
LRRK2 Roc-COR domain mutations interfere with axonal transport. Kymographs show transport of (**a**) DsRed-labelled mitochondria in rat cortical neurons transfected to express the indicated acGFP1-tagged LRRK2 variants (Control is expressing acGFP1 alone) or (**b**) mito-GFP-labelled mitochondria in transgenic *Drosophila* motor neurons expressing the indicated LRRK2 variants. Charts are mean±s.e.m. of quantified mitochondrial transport shown as percentage of total mitochondria. (**c**) Locomotion assays for climbing and flight behaviour of LRRK2 variants expressed in motor neurons by *D42-GAL4*. *Drosophila* control is driver/reporter crossed to a *lacZ* transgene. *P<0.05, **P<0.01, ***P<0.001, ****P<0.0001, one-way analysis of variance followed by Fisher’s least significant difference *post hoc* test. (**a**) *N* (cells; from four to eight experiments)=Ctrl, 5; WT, 12; R1441C, 6; Y1699C, 20; G2019S, 7. (**b**) *N* (animals)=Ctrl, 10; WT, 10; R1441C, 12; Y1699C, 14; G2019S, 11. (**c**) *N* (animals)=Ctrl, 95; WT, 141; R1441C, 93; Y1699C, 122; G2019S, 59.

To study the effect of pathogenic LRRK2 mutations on axonal transport, we expressed wild-type (WT) or mutant (R1441C, Y1699C or G2019S) LRRK2 variants in rat cortical neuron cultures, and analysed the movement of mitochondria from time-lapse recordings. Whereas the expression of LRRK2-WT or LRRK2-G2019S did not affect mitochondrial transport, both LRRK2-R1441C and LRRK2-Y1699C significantly inhibited the transport of mitochondria by disrupting both anterograde and retrograde motility (Fig. 1a). Detailed analysis revealed that both LRRK2-R1441C and LRRK2-Y1699C decreased anterograde and retrograde transport frequencies, with a concomitant increase in the duration of pauses and decreased velocities (Supplementary Fig. 1).

To investigate if this axonal transport defect occurred *in vivo*, we analysed the transport of mitochondria in motor neurons of *Drosophila* larvae expressing human LRRK2 variants. Similar to mammalian neurons, the expression of LRRK2-WT or LRRK2-G2019S did not adversely affect mitochondrial transport, whereas the LRRK2-R1441C or LRRK2-Y1699C mutants reduced the number of motile mitochondria in both anterograde and retrograde directions (Fig. 1b). Moreover, the effects are evolutionarily conserved as the expression of *Drosophila* Lrrk-R1069C (equivalent to LRRK2-R1441C) or Lrrk-Y1383C (equivalent to LRRK2-Y1699C) inhibited both anterograde and retrograde transport, while the expression of Lrrk-WT similarly had no effect on transport (Supplementary Fig. 2). Together these results demonstrate that the expression of pathogenic Roc-COR variants of LRRK2 or its *Drosophila* homologue Lrrk inhibit bidirectional axonal transport of mitochondria *in vitro* and *in vivo*.

The *Drosophila* model enabled us to investigate whether the aberrant axonal transport resulted in a functional deficit of the motor system. Adult flies expressing variant forms of LRRK2 or Lrrk in motor neurons were assayed for climbing and flight ability. Flies expressing WT LRRK2/Lrrk or LRRK2-G2019S showed no defect in locomotion compared with controls, consistent with a lack of effect on axonal transport (Fig. 1c and Supplementary Fig. 2). However, LRRK2-R1441C and LRRK2-Y1699C caused a significant decrease in both climbing and flight ability (Fig. 1c). Consistent with a conserved effect of these mutations, Lrrk-R1069C and Lrrk-Y1383C also caused locomotor deficits (Supplementary Fig. 2).

### Roc-COR mutants form filamentous structures on microtubules

**Figure 2:**
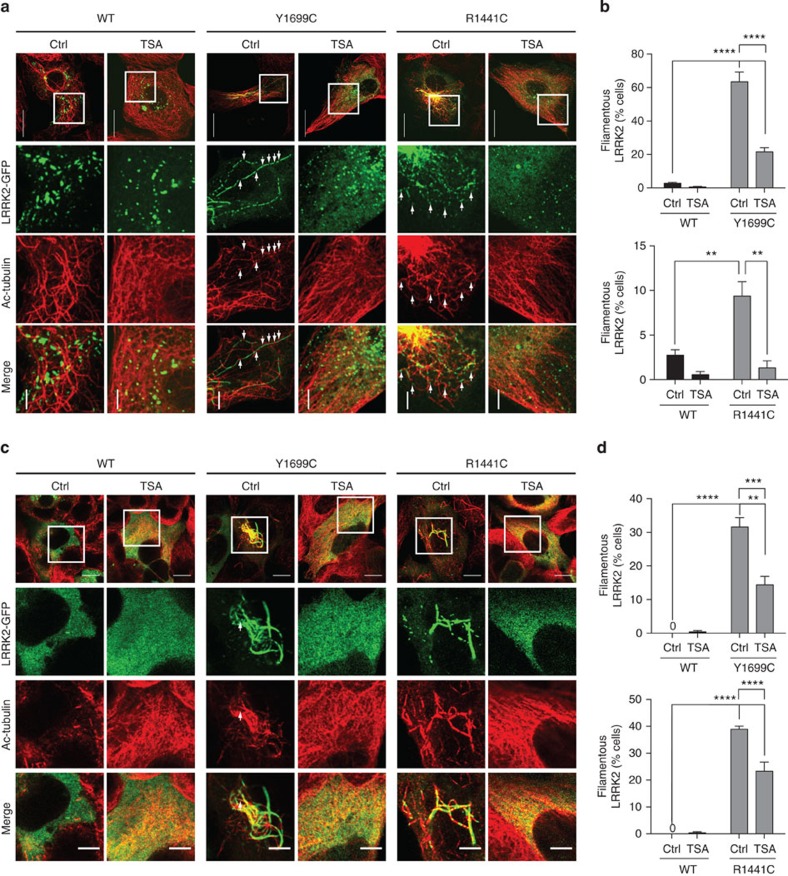
LRRK2 Roc-COR domain mutants form TSA-sensitive filamentous structures. CV1 (**a**) or HEK293 (**c**) cells were transfected with acGFP1-tagged LRRK2 variants (green) as indicated. Twenty-four hours after transfection, the cells were treated with vehicle (Ctrl) or 0.5 μM TSA for 4 h. After treatment, the cells were fixed and processed for immunostaining using anti-acetylated α-tubulin antibody (red). Scale bar, 20 μm (**a**), 10 μm (**c**); zoom, 5 μm. The percentage of vehicle (Ctrl) and TSA-treated (TSA) CV1 (**b**) and HEK293 (**d**) cells exhibiting filamentous LRRK2 was quantified (mean±s.e.m., zero values are indicated (0)). **P<0.01, ***P<0.001, ****P<0.0001, two-way analysis of variance followed by Fisher’s least significant difference *post hoc* test. (**b**) *N* (coverslips counted, ~50 cells per coverslip; from four experiments)=WT, 7; WT+TSA, 5; Y1699C, 4; Y1699C+TSA, 5; R1441C, 11; R1441C+TSA, 4. (**d**) *N* (coverslips counted, ~50 cells per coverslip; from four experiments)=WT, 5; WT+TSA, 5; Y1699C, 13; Y1699C+TSA, 5; R1441C, 15; R1441C+TSA, 5.

The bidirectional nature of the axonal transport defect suggested the disturbance of a common transport component such as microtubules. It has been reported that LRRK2 can interact with microtubules and even alter tubulin acetylation^14^,^15^,^16^,^17^,^18^. Importantly, the acetylation status of microtubules is known to influence the rate of transport along them, with increased acetylation promoting axonal transport^25^,^26^. Therefore, we hypothesized that LRRK2 Roc-COR mutants may affect the equilibrium of microtubules. To address this, we analysed the association of LRRK2 variants with microtubules in mammalian CV1 and HEK293 cells, focusing specifically on acetylated microtubules. In contrast to LRRK2-WT, we found that LRRK2-R1441C and LRRK2-Y1699C had a propensity to form filamentous structures that appeared to align with microtubules (Fig. 2). These structures are reminiscent of previously reported skein-like LRRK2 structures^17^,^27^. Furthermore, where LRRK2-R1441C or LRRK2-Y1699C was present, α-tubulin acetylation was lacking. These observations are consistent with LRRK2-R1441C and LRRK2-Y1699C inhibiting axonal transport by interfering with microtubule acetylation.

### Increasing microtubule acetylation inhibits LRRK2 filaments

We next investigated whether the interaction of LRRK2 variants with deacetylated microtubules was reversible and sensitive to microtubule acetylation. We treated CV1 and HEK293 cells with the broad deacetylase inhibitor trichostatin A (TSA), which promotes α-tubulin acetylation^26^, and analysed filament formation. In both CV1 and HEK293 cells, TSA treatment significantly reduced the formation of filamentous LRRK2 (Fig. 2)

**Figure 3:**
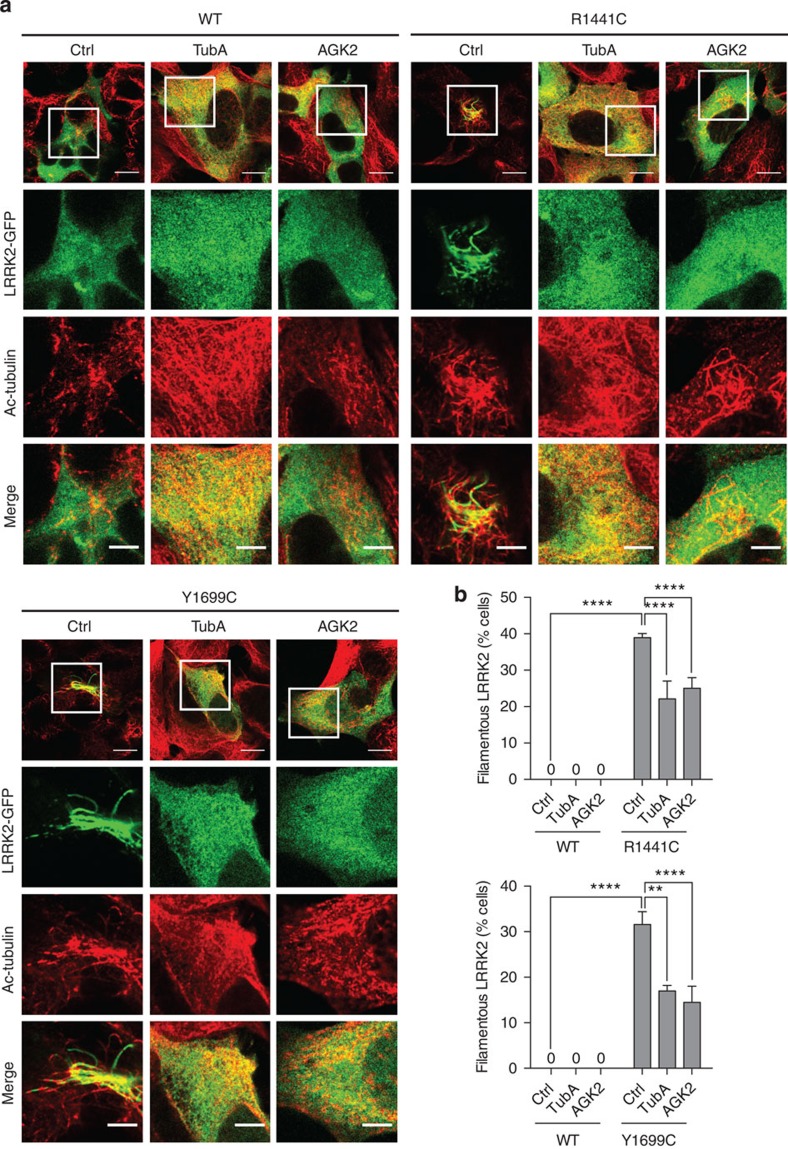
Both HDAC6 and SIRT2 inhibitors prevent LRRK2 Roc-COR mutant filamentous structures. (**a**) HEK293 cells were transfected with acGFP1-tagged LRRK2 variants (green) as indicated. Twenty-four hours after transfection, the cells were treated with vehicle (Ctrl), 2 μM tubastatin-A (TubA) or 5 μM AGK2 for 4 h. After treatment, the cells were fixed and processed for immunostaining using anti-acetylated α-tubulin antibody (red). Scale bar, 10 μm; zoom, 5 μm. (**b**) The percentage of vehicle (Ctrl), TubA and AGK2-treated HEK293 cells exhibiting filamentous LRRK2 was quantified (mean±s.e.m., zero values are indicated (0)). **P<0.01, ****P<0.0001, two-way analysis of variance followed by Fisher’s least significant difference *post hoc* test. *N* (coverslips counted, ~50 cells per coverslip, from four experiments)=WT, 5; WT+TubA, 5; WT+AGK2, 5; Y1699C, 13; Y1699C+TubA, 4; Y1699C+AGK2, 9; R1441C, 15; R1441C+TubA, 5; R1441C+AGK2, 9.

The family of histone deacetylases (HDACs) is classified into four groups based on function and DNA sequence similarity. Classes I, IIa and IIb are considered the classical HDACs whose activities are inhibited by TSA. Class III comprises a family of NAD^+^-dependent proteins called the Sirtuins (SIRTs) that are not affected by TSA. Class IV is an atypical class based solely on DNA sequence similarity to the others. Both the class IIb member HDAC6 and class III member SIRT2 have been shown to deacetylate α-tubulin^28^,^29^ and conversely their inhibition increases microtubule acetylation. Thus, although in our assays HDAC6 was likely to be the main target of TSA, other pathways could have been affected by TSA treatment. To more selectively target microtubule acetylation, we treated HEK293 cells expressing LRRK2 Roc-COR mutants with the specific HDAC6 inhibitor tubastatin-A^30^,^31^,^32^ or the SIRT2-specific inhibitor AGK2 (ref. 33). Specific inhibition of either HDAC6 or SIRT2 prevented the formation of filamentous LRRK2 to the same extend as TSA (Fig. 3).

**Figure 4:**
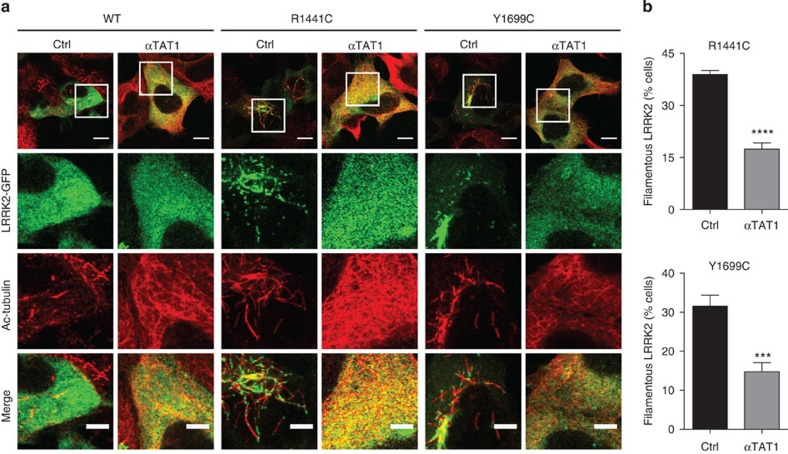
αTAT1 expression prevents LRRK2 Roc-COR mutant filamentous structures. (**a**) HEK293 cells were co-transfected with acGFP1-tagged LRRK2 variants (green) and either empty vector (Ctrl) or αTAT1 as indicated (3 × excess of empty vector or αTAT1 was used to ensure co-transfection). Twenty-four hours after transfection, the cells were fixed and processed for immunostaining using anti-acetylated α-tubulin antibody (red). Scale bar, 10 μm; zoom, 5 μm. (**b**) The percentage of empty vector- (Ctrl) and αTAT1-transfected HEK293 cells exhibiting filamentous LRRK2 was quantified (mean±s.e.m.). ***P<0.001, ****P<0.0001, Student’s *t*-test, *N* (coverslips counted, ~50 cells per coverslip; from four experiments)=WT, 5; WT+αTAT1, 5; Y1699C, 13; Y1699C+αTAT1, 13; R1441C, 15; R1441C+αTAT1, 13.

To further characterize the potential role of microtubule acetylation in the LRRK2 Roc-COR mutant filamentous phenotype, we expressed the α-tubulin acetyl transferase αTAT1 (also called MEC17)^34^,^35^. Because α-tubulin is the major cellular substrate of αTAT1, the overexpression of αTAT1 specifically increases microtubule acetylation^36^. αTAT1 expression also significantly reversed the filamentous LRRK2 Roc-COR mutant phenotype (Fig. 4) confirming that microtubule acetylation regulates the association of LRRK2 Roc-COR mutants with microtubules.

Together these findings support previous reports that LRRK2 associates with microtubules and indicate that LRRK2 Roc-COR mutants interfere with α-tubulin acetylation, which can be restored by the inhibition of the deacetylase enzymes HDAC6 and SIRT2 or by the overexpression of αTAT1.

### TSA restores axonal transport and locomotion

**Figure 5:**
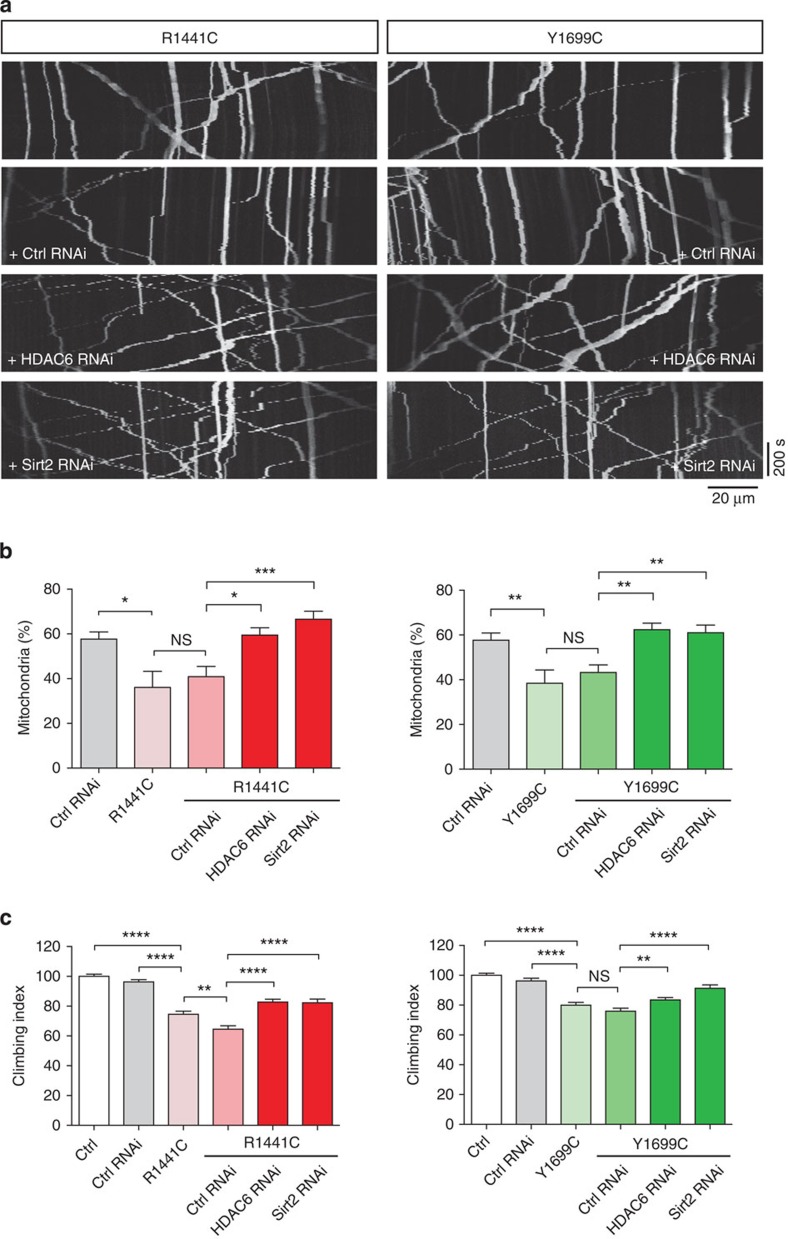
*HDAC6* and *Sirt2* RNAi restores axonal transport and locomotion. (**a**) Kymographs of mitochondria in transgenic *Drosophila* motor neurons expressing mito-GFP with indicated LRRK2 variants alone (top) or in combination with *HDAC6* or *Sirt2* RNAi transgenes. Ctrl RNAi is expressing a *lacZ-RNAi* transgene. (**b**) Charts are mean±s.e.m. of quantified mitochondrial transport shown as percentage of total mitochondria. (**c**) Locomotion assays for climbing behavior of indicated genotypes. Transgene expression is driven by *D42-GAL4*. Ctrl is driver crossed to a *lacZ* transgene. *P<0.05, **P<0.01, ***P<0.001, ****P<0.0001, one-way analysis of variance followed by Fisher’s least significant difference *post hoc* test. (**b**) left chart: *N* (animals)=Ctrl RNAi, 11; R1441C, 9; R1441C+Ctrl RNAi, 23; R1441C+HDAC6 RNAi, 13; R1441C+Sirt2 RNAi, 12; right chart: *N* (animals)=Ctrl RNAi, 11; Y1699C, 11; Y1699C+Ctrl RNAi, 13; Y1699C+HDAC6 RNAi, 15; Y1699C+Sirt2 RNAi, 18. (**c**) left chart: *N* (animals)=Ctrl, 379; Ctrl RNAi, 326; R1441C, 367; R1441C+Ctrl RNAi, 245; R1441C+HDAC6 RNAi, 300; R1441C+Sirt2 RNAi, 218; right chart: *N* (animals)=Ctrl, 329; Ctrl RNAi, 281; Y1699C, 381; Y1699C+Ctrl RNAi, 338; Y1699C+HDAC6 RNAi, 413; Y1699C+Sirt2 RNAi, 170.

Reversal of the association of LRRK2 Roc-COR mutants with deacetylated microtubules prompted us to address whether modulating microtubule acetylation could also reverse the axonal transport defects *in vitro* and *in vivo*. We first targeted the tubulin deacetylases *in vivo* using transgenic *Drosophila HDAC6* or *Sirt2* RNA interference (RNAi) lines. Loss of either *HDAC6* or *Sirt2* effectively restored axonal transport of mitochondria in LRRK2 Roc-COR mutant transgenic flies (Fig. 5a,b). Moreover, the knockdown of *HDAC6* or *Sirt2* was able to significantly rescue climbing and flight deficits caused by mutant LRRK2 (Fig. 5c).

**Figure 6:**
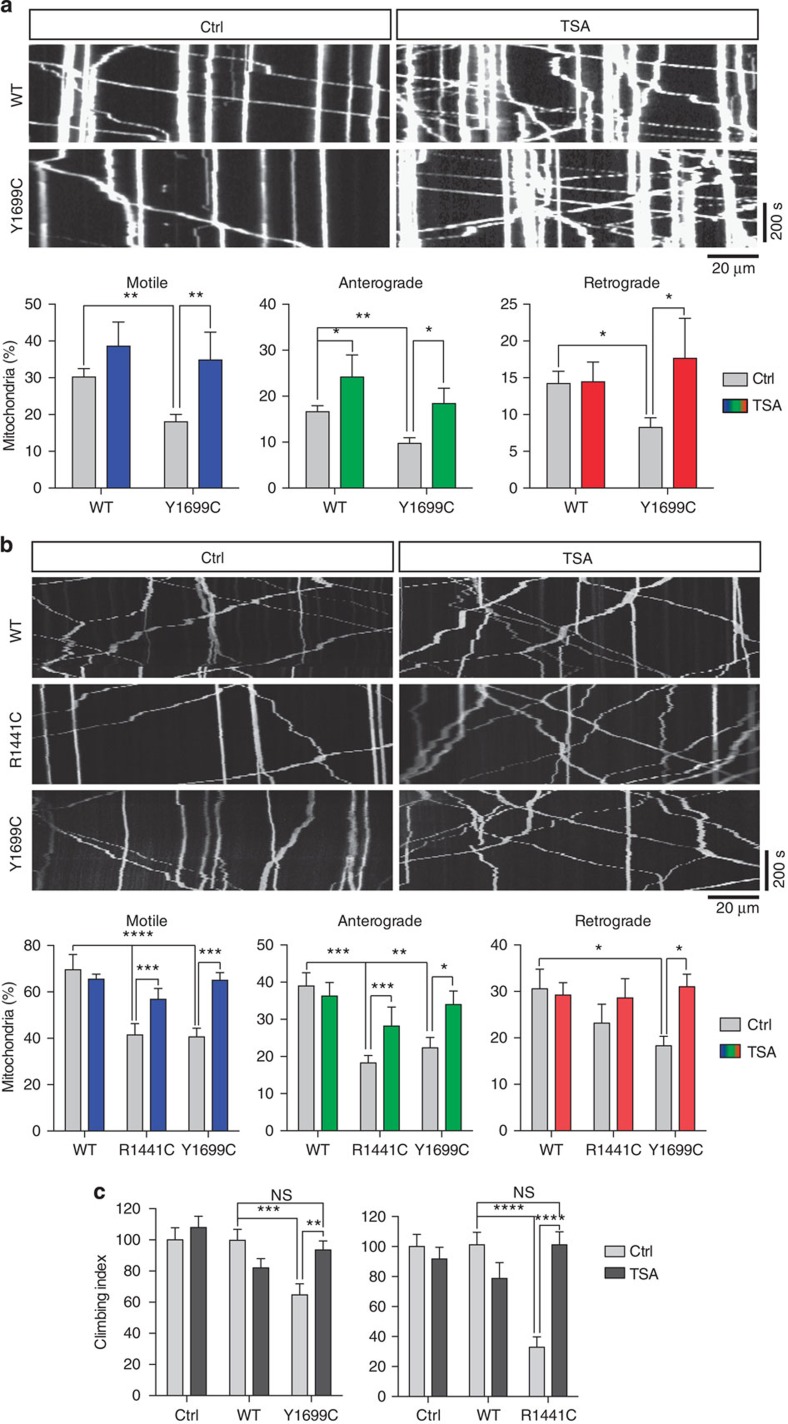
Axonal transport and locomotion is restored by TSA treatment. (**a**) Kymographs show transport of DsRed-labelled mitochondria in rat cortical neurons transfected with acGFP1-tagged LRRK2 variants as indicated and treated with vehicle (Ctrl) or 0.5 μM TSA for 4 h. Charts are mean±s.e.m. of quantified mitochondrial transport shown as percentage of total mitochondria. (**b**) Kymographs of mitochondria in transgenic *Drosophila* motor neurons expressing mito-GFP and indicated LRRK2 variants. Ctrl is expressing a *lacZ* transgene. *Drosophila* were raised on food containing vehicle control (Ctrl) or 10 μM TSA. Charts are mean±s.e.m. of quantified mitochondrial transport shown as percentage of total mitochondria. (**c**) Locomotion assays for climbing behavior following vehicle control or TSA treatment. *Drosophila* were raised on normal food, then fed vehicle (Ctrl) or TSA supplemented food for 5 days before testing. Transgene expression is driven by *D42-GAL4*. *P<0.05, **P<0.01, ***P<0.001, ****P<0.0001, two-way analysis of variance followed by Fisher’s least significant difference *post hoc* test. (**a**) *N* (cells; from three to five experiments)=WT Ctrl, 30; WT TSA, 7; Y1699C Ctrl, 20; Y1699C TSA, 5; (**b**) *N* (animals)=WT Ctrl, 10; WT+TSA, 10; R1441C Ctrl, 13; R1441C+TSA, 13; Y1699C Ctrl, 11; Y1699C+TSA, 11; (**c**), right chart: *N* (animals)=Ctrl Ctrl, 84; Ctrl+TSA, 77; WT Ctrl, 68; WT+TSA, 46; R1441C Ctrl, 43; R1441C+TSA, 53; left chart: *N* (animals)=Ctrl Ctrl, 41; Ctrl+TSA, 45; WT Ctrl, 56; WT+TSA, 78; Y1699C Ctrl, 59; Y1699C+TSA, 87.

The ability of TSA to prevent mutant LRRK2 from aberrant binding to microtubules makes this an attractive therapeutic intervention. We tested this idea by treating primary rat neurons expressing LRRK2-Y1699C with TSA. Encouragingly, this was able to restore axonal transport to control levels (Fig. 6a). To extend this observation *in vivo*, *Drosophila* expressing the LRRK2 variants were raised on TSA-dosed food. Remarkably, this completely rescued the transport defects caused by LRRK2-R1441C or Y1699C (Fig. 6b). Similarly, transport defects caused by Lrrk-R1069C or Y1383C expression were also restored by feeding TSA (Supplementary Fig. 3). Thus, both genetic and pharmacological approaches *in vitro* and *in vivo* show that increasing microtubule acetylation can rescue LRRK2 Roc-COR mutant-induced axonal transport defects.

Finally, we asked whether treatment with TSA would be able to rescue the locomotor deficits cause by the defective axonal transport even after the onset of the phenotype. We took newly emerged flies expressing WT and mutant forms of LRRK2 or Lrrk that had been raised on normal food, which as we previously showed already exhibited climbing and flight deficits (Fig. 1c). These young adult flies were then fed TSA for 5 days and tested for locomotion. Notably, even with this modest increase in age, the locomotor deficit had worsened in control-fed animals (Fig. 6c and Supplementary Fig. 3c). However, the administration of TSA was sufficient to fully restore locomotor ability in the LRRK2-R1441C- or Y1699C- and Lrkk-R1069C- or Y1383C-expressing animals (Fig. 6c and Supplementary Fig. 3c).

These results demonstrate that increasing microtubule acetylation either genetically by knockdown of deacetylase enzymes or by systemic administration of a deacetylase inhibitor is sufficient to restore a primary cellular defect and reverse the associated behavioural disruption, even after the onset of behavioural symptoms.

## Discussion

Disruption of axonal transport is an early feature in a number of neurodegenerative diseases including PD^19^. Analysis of axonal transport of WT and pathogenic mutants of α-synuclein have revealed reduced transport rates of the mutants, which may contribute to the formation of Lewy bodies^21^. PINK1 and Parkin, mutations in which cause the majority of early-onset recessive familial PD^37^,^38^, have been shown to directly affect mitochondrial transport^23^,^24^ and this in turn regulates mitophagy^39^. Halting of axonal transport has also been observed in mitochondrial neurotoxin models of sporadic PD^40^,^41^,^42^.

In this study, we investigated whether PD-associated mutations in LRRK2 affect axonal transport and cause dysfunction of the motor system. We found that pathogenic mutations in the Roc-COR domain of LRRK2 (R1441C and Y1699C) disrupt axonal transport of mitochondria *in vitro* in cultured neurons and *in vivo*, and cause locomotor deficits in *Drosophila*. The effects are evolutionarily conserved since the expression of mutant *Drosophila Lrrk* variants also inhibited transport and motor ability.

LRRK2 has been shown to interact with microtubules but the physiological relevance of this interaction is poorly understood^14^,^15^,^16^,^17^,^18^. We found that both LRRK2-R1441C and Y1699C mutants, but not LRRK2-WT, formed filament-like structures that decorated deacetylated microtubules. Previous work has shown that acetylation of microtubules regulates axonal transport^25^,^26^ and has implicated LRRK2 in the regulation of microtubule acetylation^18^. We therefore increased microtubule acetylation using pharmacological inhibitors or RNAi knockdown of the deacetylase enzymes HDAC6 and SIRT2, or by the expression of αTAT1 and found that this prevented the decoration of microtubules by LRRK2-R1441C and Y1699C. Importantly, increasing microtubule acetylation restored axonal transport to WT levels *in vitro* and *in vivo*.

Interestingly the kinase domain mutant LRRK2-G2019S did not affect axonal transport. Several groups have shown that the R1441C and Y1699C mutations inhibit the GTPase activity of LRRK2 but have normal kinase activity^6^,^7^,^43^,^44^, whereas LRRK2-G2019S has increased kinase activity but normal GTPase activity compared with WT LRRK2 (refs 8, 9, 45, 46). Thus, our results suggest that the inhibition of axonal transport is likely linked to decreased LRRK2 GTPase activity. This is in line with increasing evidence suggesting that the GTPase activity of LRRK2 plays an important role in pathogenesis^47^. Interestingly, the pharmacological inhibition of LRRK2 kinase activity has been shown to induce a redistribution of LRRK2 that appears similar to the microtubule-attached distribution we observed with LRRK2 Roc-COR mutants^48^ and the binding of LRRK2 to tubulin was significantly enhanced by LRRK2 kinase inhibition in an indirect reporter assay^16^. However, since the LRRK2 Roc-COR mutants analysed here have normal kinase activity^6^,^7^,^43^,^44^, our data suggest that the GTPase activity of LRRK2 plays a key role in LRRK2’s interaction with microtubules. The effect of LRRK2 kinase inhibitors may be explained by a model in which LRRK2 kinase regulates the GTPase via phosphorylation^47^.

The *Drosophila* model allowed us to extend our observations of defective axonal transport in a number of ways. First, it allowed us to address whether the inhibition of axonal transport by LRRK2 Roc-COR mutants had any physiological consequences in adult motor behaviours. We found that *Drosophila* expressing LRRK2 Roc-COR mutants also resulted in a deficit of the motor system, causing a decrease in both climbing and flight ability. Furthermore, when we restored axonal transport by increasing microtubule acetylation using *HDAC6* or *Sirt2* RNAi, this also restored normal locomotor ability. Thus, these results argue that inhibition of axonal transport can cause functional deficits *in vivo*.

Second, this system also enabled us to assess whether pharmacological restoration of acetylated microtubules could reverse the locomotor phenotype after the onset of locomotor symptoms. Indeed, we found that the systemic administration of TSA was able to fully restore locomotor ability, showing that the effects of the LRRK2 Roc-COR mutants are reversible and, at least in the fly, are amenable to *post hoc* treatment. Thus, our study adds PD to the growing list of neurodegenerative diseases that involve disturbed axonal transport in which deacetylase inhibitors have been shown to confer protection including Charcot*–*Marie–Tooth disease, Alzheimer’s disease, amyotrophic lateral sclerosis and Huntington’s disease^26^,^49^,^50^,^51^,^52^,^53^,^54^,^55^.

In conclusion, our results reveal that mutant LRRK2-associated PD involves defective axonal transport and suggest that treating with deacetylase inhibitors to restore transport may have therapeutic potential.

## Methods

### Plasmids

pAcGFP1-LRRK2-WT, Y1699C and R1441C variants were a gift from M. Cookson (NIH, Bethesda). pAcGFP1-LRRK2-G2019S was generated by mutagenesis of pAcGFP1-LRRK2-WT using a QuikChange Site-Directed Mutagenesis Kit (Stratagene) according to the manufacturer’s instructions. Mutagenic primers were: 5′-caaagattgctgactacagcattgctcagtactgctg-3′ (forward) and 5′-cagcagtactgagcaatgctgtagtcagcaatctttg-3′ (reverse). Mutagenesis was verified by sequencing. The pSPORT6-MEC17 (αTAT1) expression plasmid has been described^35^ and was a gift from J. Gaertig (University of Georgia, Athens, GA).

### Cell culture and plasmid transfection

CV1 and HEK293 cells obtained from the ATCC were maintained in Dulbecco’s modified Eagle’s medium (Invitrogen) containing 4.5 g l^−1^ glucose, 10% foetal bovine serum (Sera Laboratories), 2 mM L-glutamine (Invitrogen) and 1 mM sodium pyruvate (Sigma) and were transfected using Exgen500 (Fermentas) or Lipofectamine 2000 (Invitrogen) according to the manufacturer’s instructions. All cells were used in experiments 16–24 h post transfection.

Cortical neurons were isolated and transfected as previously described^56^,^57^,^58^,^59^. Briefly, cortical neurons were isolated from embryonic day 18 rat embryos and cultured on glass coverslips coated with poly-L-lysine in six or 12-well plates in neurobasal medium containing B27 supplement (Invitrogen), 100 IU ml^−1^ penicillin, 100 μg ml^−1^ streptomycin and 2 mM L-glutamine. Cells were cultured for 7 days and then transfected using a calcium phosphate Profection kit (Promega). For studies on the effects of LRRK2 on axonal transport, neurons were transfected with twice the amount of LRRK2 plasmid compared with DsRed-Mito to ensure that all cells visualized for mitochondrial transport expressed LRRK2 and this was confirmed by AcGFP1 fluorescence.

### Antibodies

Anti-acetylated α-tubulin mouse monoclonal antibody was from Sigma (clone 6-11B-1) and used diluted 1:5,000. Secondary antibodies were Alexa fluorophore(546 and 633)-coupled goat anti-mouse IgG from Invitrogen (dilution 1:500).

### Immunofluorescence analysis of LRRK2 microtubule interaction

Immunostaining was performed as described previously^60^. Briefly, CV1 or HEK293 cells on glass coverslips were fixed with 3.7% formaldehyde in PBS for 20 min at room temperature. After washing with PBS, residual formaldehyde was quenched by incubation with 50 mM NH_4_Cl in PBS for 15 min at room temperature, followed by a second round of washing with PBS. Subsequently, the cells were permeabilized with −20 °C MeOH for 20 min or by incubation with 0.2% Triton-X-100 in PBS for 3 min. MeOH or Triton-X-100 was removed by washing with PBS.

After fixing, the cells were incubated with PBS containing 0.2% fish gelatine (PBS/F) for 30 min at room temperature and then with the primary antibody in PBS/F for 1 h. After washing with PBS/F, the cells were incubated with secondary antibody in PBS/F for 45 min at room temperature. After a final wash, the samples were mounted in Moviol (Calbiochem) containing 1% DABCO or Dako fluorescence mounting medium.

Confocal images were recorded with an LSM510Meta (Carl Zeiss) or a Leica SP5 (Leica Microsystems) confocal microscope using a × 63, 1.4NA objective.

### Axonal transport of mitochondria in rat cortical neurons

Live microscopy of mitochondrial axonal transport in cortical neurons was performed with an Axiovert S100 microscope (Carl Zeiss) equipped with a Lambda LS Xenon-Arc light source (Sutter Instrument Company, Novato, CA), an EGFP/DsRed filterset (Chroma Technology Corp.), × 40 EC Plan-Neofluar 1.3 N.A. objective (Zeiss), Lambda 10-3 filter wheel (Sutter Instrument Co.) and a Photometrics Cascade-II 512B High Speed EMCCD camera (Photometrics). Post transfection (36–48 h), neurons on coverslips were transferred to a custom observation chamber mounted on the stage of the microscope. The cells were maintained at 37 °C using an objective heater (Tempcontrol 37-2, Carl Zeiss) and ‘The Box’ Microscope Temperature Control System (Life Imaging Systems). Mitochondrial movements were recorded for 10 min with 3 s time-lapse interval using Metamorph software (Molecular Devices).

Image analysis was performed with ImageJ developed by Wayne Rasband (NIH, Bethesda, USA; http://rsb.info.nih.gov/ij/) extended with custom plug-ins, or Metamorph. Calculations of mitochondrial transport parameters were as described before^57^,^59^ except that the overall transport of mitochondria in Figs 1 and 5 were analysed from kymographs using the SlopeToVelocity plugin described previously^59^. Briefly, this plugin calculates the velocity by measuring the distance between the position of individual mitochondria at the start and end of time-lapse recordings and dividing by the time elapsed. This yields an overall velocity of transport that includes anterograde and retrograde movements and stationary periods. Mitochondria were subsequently classified as motile (velocity >0.1 μm s^−1^) or stationary (velocity ≤0.1 μm s^−1^).

### *Drosophila* stocks and procedures

*Drosophila* were raised under standard conditions at 25 °C on agar, cornmeal and yeast food. Transgenic lines used were: UAS-*LRRK2* WT^61^, UAS-*LRRK2* R1441C^62^, UAS-*LRRK2* Y1699C^63^, UAS-*LRRK2* G2019S^61^, UAS-*Lrrk* WT^64^, UAS-*Lrrk* R1069C^64^ and UAS-*Lrrk* Y1383C^63^. *D42-GAL4, CCAP-GAL4,* UAS*-mitoGFP* and UAS-*lacZ* were obtained from the Bloomington Drosophila Stock Center. UAS-*lacZ*-RNAi (#51446), UAS-*HDAC6*-RNAi (#108831) and UAS-*Sirt2*-RNAi (#21999) were obtained from the Vienna Drosophila Resource Centre^65^. Flight and climbing assays were performed as previously described^66^.

### Dissection and imaging of axonal transport in *Drosophila*

Live dissections of wandering third instar larvae were performed as previously described^67^. Individual larvae were placed on Sylgard slide and pinned on both ends with the dorsal side up. Larval body wall muscles were carefully opened using microdissection scissors. Internal organs were removed with forceps without disturbing the ventral ganglion and motor neurons. The cuticle was laterally expanded on both sides and pinned. The whole preparation was washed in 1 ml dissecting buffer containing 128 mM NaCl, 1 mM EGTA, 4 mM MgCl_2_, 2 mM KCl, 5 mM HEPES and 36 mM sucrose, pH 7.2. The buffer was replaced and the larvae kept immersed in buffer during imaging. Imaging was done on an Olympus FV1000 confocal using × 60 water immersion objective (NA 0.90, Olympus LUMPLFL). Time-lapse movies were acquired at the rate of 1 frame per 5 s for 500 s. A minimum of 10 movies (one movie per larva) were taken for each genotype/treatment.

### Analysis of mitochondrial movement in *Drosophila*

All movies were analysed using ImageJ software. The movies were processed in ImageJ and kymographs were generated as previously described^59^. Using kymographs, the number of mitochondria moving in anterograde and retrograde directions were quantified along with the number of stationary mitochondria. Mitochondria were scored as moving if the organelle is in motion for at least 60% of the movie. All scoring was performed blind to the experimental condition.

### Drug treatment in *Drosophila*

Crosses were raised in standard medium supplemented with 10 μM TSA (Sigma-Aldrich T8552). Dimethylsulphoxide (0.06%) was used as a control. For adult climbing, crosses with *D42-GAL4* were raised in standard medium and young progeny of 1–2 days were collected. Flies were starved in empty vials for 3–4 h at 25 °C and then placed in vials with filter papers soaked with 1 ml solution containing 10% sucrose and 100 μM TSA^68^. Dimethylsulphoxide (0.6%) was used as a control. The filter papers were checked every day and replaced after 48 h. Climbing assays were performed after 5 days, that is, animals were 6–7 days old.

### Statistical analysis

Calculations and statistical analysis were performed using Excel (Microsoft Corporation, Redmond, WA), and Prism software (GraphPad Software, Inc., San Diego, CA). Statistical significance was determined by one-way or two-way analysis of variance (ANOVA) followed by Fisher’s least significant difference *post hoc* test or by *t*-test as indicated in the figure legends.

## Additional information

**How to cite this article:** Godena, V. K. *et al.* Increasing microtubule acetylation rescues axonal transport and locomotor deficits caused by LRRK2 Roc-COR domain mutations. *Nat. Commun.* 5:5245 doi: 10.1038/ncomms6245 (2014).

## Supplementary Material

Supplementary Information Supplementary Figures 1-3
