# Supplementary material for: Increasing microtubule acetylation rescues axonal transport and locomotor deficits caused by LRRK2 Roc-COR domain mutations
Source: Nat Commun. 2014 Oct 15;5:5245. doi: 10.1038/ncomms6245 (PMC4208097; doi:10.1038/ncomms6245)
Supplement: Supplementary Information — Supplementary Figures 1-3 [file ncomms6245-s1.pdf]

Increasing Microtubule Acetylation Rescues Axonal Transport And Locomotor Deficits Caused by LRRK2 Roc-COR Domain Mutations

Vinay K. Godena[1,3,4,†], Nicholas Brookes-Hocking[5,†], Annekathrin Moller[2], Gary Shaw[2], Matthew Oswald[1,3,6], Rosa M. Sancho[5,7], Christopher C.J. Miller[5], Alexander J. Whitworth[1,3,4,*], Kurt J. De Vos[2,4,*].

Supplementary information

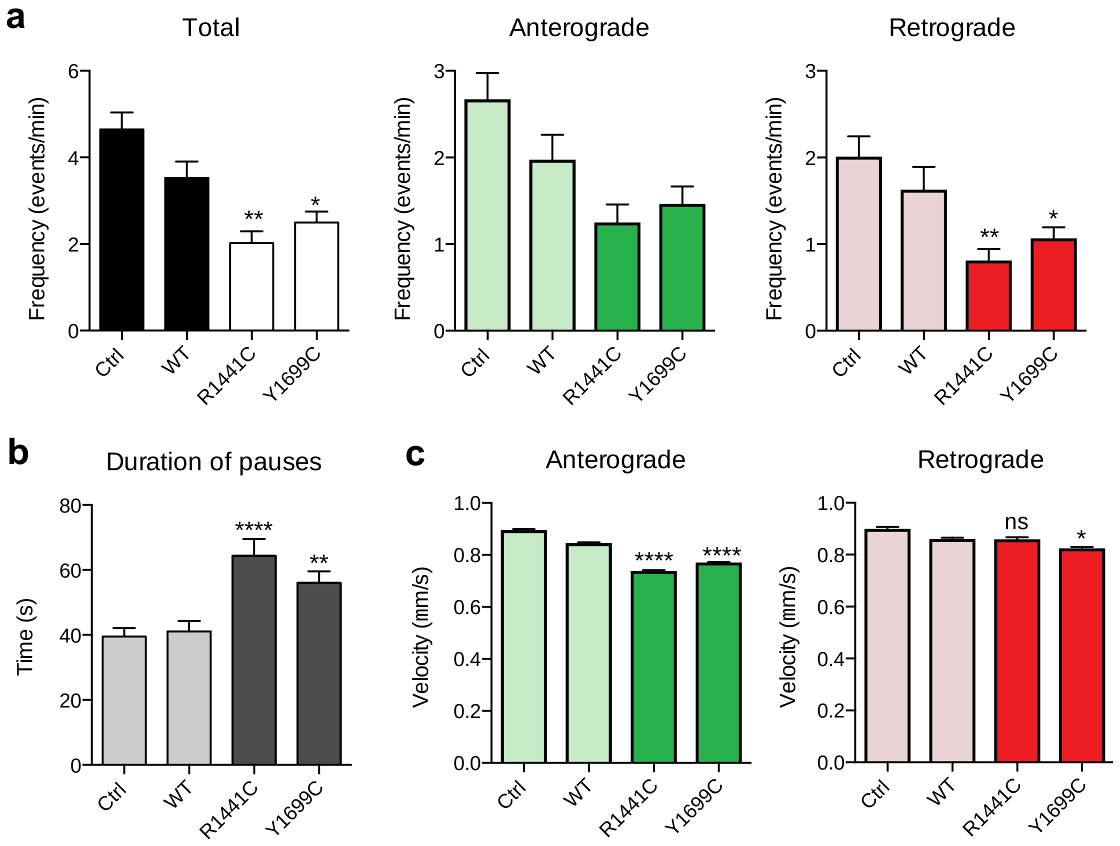

Supplementary Fig. 1. Analysis of mitochondrial axonal transport parameters in rat cortical neurons expressing LRRK2 variants. (**a**) Frequency of movements expressed as events per minute for individual mitochondria. (**b**) Duration of pauses. (**c**) Velocity of single movement events. (**a**) N (mitochondria) = 208, 187, 245, 318; (**b**) N (events) = 1285, 802, 636, 1032; (**c**) N (events) = 1644, 1547, 1108, 1570. * P < 0.05, ** P < 0.01, *** P < 0.001, **** P < 0.0001, one-way ANOVA with Fisher's LSD post-hoc test.

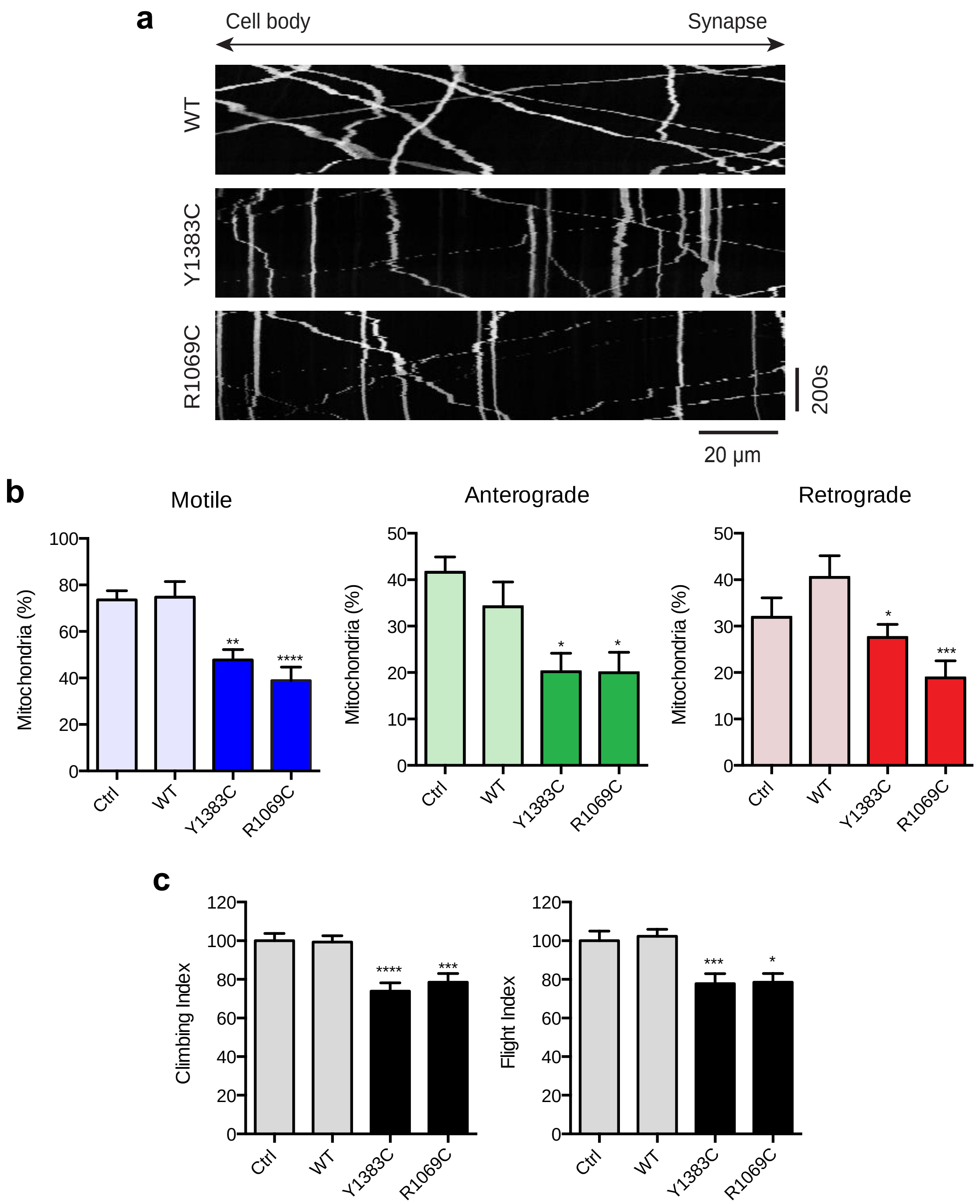

Supplementary Fig. 2. Expression of *Drosophila* Lrrk transgenes with Roc-COR mutations inhibits axonal transport and locomotor ability. (**a**) Kymographs of mitochondria in Drosophila motor neurons expressing mito-GFP and indicated Lrrk variants. (**b**) Charts are mean ± s.e.m. of quantified mitochondrial transport shown as percentage of total mitochondria. N (animals) = Ctrl: 10; WT: 9; R1069C: 11; Y1383C: 11 (**c**) Locomotion assays for climbing and flight behavior of motor neuron expressed Lrrk variants. Control is driver/reporter crossed to a lacZ transgene. N (animals) = Ctrl: 95; WT: 120; R1069C: 98; Y1383C: 104. * $P < 0.05$, ** $P < 0.01$, *** $P < 0.001$, **** $P < 0.0001$, one-way ANOVA with LSD post-hoc test.

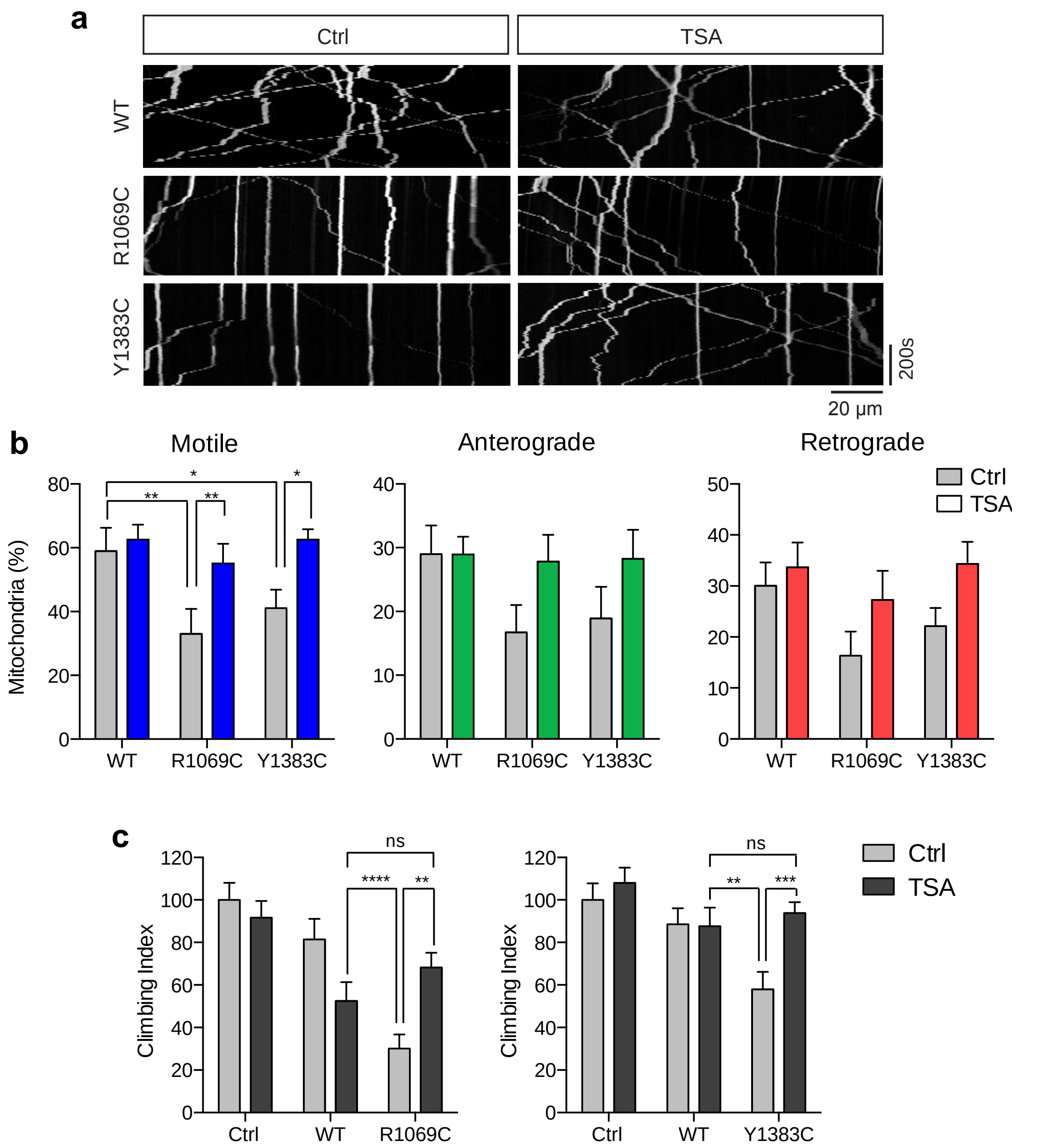

Supplementary Fig. 3. Axonal transport and locomotion caused by mutant Lrrk is restored by TSA treatment. (**a**) Kymographs of mitochondria transport in Drosophila motor neurons expressing mito-GFP and indicated Lrrk variants. Drosophila were raised on food containing vehicle control (ctrl; black bars) or 10 μM TSA (white bars). (**b**) Charts are mean ± s.e.m. of quantified mitochondrial transport shown as percentage of total mitochondria. N (animals) = WT Ctrl: 10; WT+TSA: 10; R1069C Ctrl: 13; R1069C+TSA: 13; Y1383C Ctrl: 11; Y1383C+TSA: 11. (**c**) Locomotion assays for climbing behavior following vehicle control or TSA treatment. Drosophila were raised on normal food, then fed vehicle (ctrl) or TSA supplemented food for 5 days before testing locomotion. Transgenic expression is driven by D42-GAL4. Control is driver/reporter crossed to a lacZ transgene. N (animals) = Ctrl Ctrl: 84; Ctrl+TSA: 77; WT Ctrl: 48; WT+TSA: 40; R1069C Ctrl: 48; R1069C+TSA: 69; Ctrl Ctrl: 41; Ctrl+TSA: 45; WT Ctrl: 52; WT+TSA: 50; Y1383C Ctrl: 43; Y1383C+TSA: 80. * $P < 0.05$, ** $P < 0.01$, *** $P < 0.001$, **** $P < 0.0001$, one-way ANOVA with LSD post-hoc test.
